# Myeloid Cell Prostaglandin E_2_ Receptor EP4 Modulates Cytokine Production but Not Atherogenesis in a Mouse Model of Type 1 Diabetes

**DOI:** 10.1371/journal.pone.0158316

**Published:** 2016-06-28

**Authors:** Sara N. Vallerie, Farah Kramer, Shelley Barnhart, Jenny E. Kanter, Richard M. Breyer, Katrin I. Andreasson, Karin E. Bornfeldt

**Affiliations:** 1 Department of Medicine, UW Diabetes Institute, University of Washington, Seattle, WA 98109, United States of America; 2 Department of Pathology, UW Diabetes Institute, University of Washington, Seattle, WA 98109, United States of America; 3 Department of Medicine, Vanderbilt University, Nashville, TN 37212, United States of America; 4 Department of Neurology and Neurological Sciences, Stanford University, Stanford, CA 94305, United States of America; Brigham and Women's Hospital, Harvard Medical School, UNITED STATES

## Abstract

Type 1 diabetes mellitus (T1DM) is associated with cardiovascular complications induced by atherosclerosis. Prostaglandin E_2_ (PGE_2_) is often raised in states of inflammation, including diabetes, and regulates inflammatory processes. In myeloid cells, a key cell type in atherosclerosis, PGE_2_ acts predominately through its Prostaglandin E Receptor 4 (EP4; *Ptger4*) to modulate inflammation. The effect of PGE_2_-mediated EP4 signaling specifically in myeloid cells on atherosclerosis in the presence and absence of diabetes is unknown. Because diabetes promotes atherosclerosis through increased arterial myeloid cell accumulation, we generated a myeloid cell-targeted EP4-deficient mouse model (EP4^M-/-^) of T1DM-accelerated atherogenesis to investigate the relationship between myeloid cell EP4, inflammatory phenotypes of myeloid cells, and atherogenesis. Diabetic mice exhibited elevated plasma PGE metabolite levels and elevated *Ptger4* mRNA in macrophages, as compared with non-diabetic littermates. PGE_2_ increased *Il6*, *Il1b*, *Il23* and *Ccr7* mRNA while reducing *Tnfa* mRNA through EP4 in isolated myeloid cells. Consistently, the stimulatory effect of diabetes on peritoneal macrophage *Il6* was mediated by PGE_2_-EP4, while PGE_2_-EP4 suppressed the effect of diabetes on *Tnfa* in these cells. In addition, diabetes exerted effects independent of myeloid cell EP4, including a reduction in macrophage *Ccr7* levels and increased early atherogenesis characterized by relative lesional macrophage accumulation. These studies suggest that this mouse model of T1DM is associated with increased myeloid cell PGE_2_-EP4 signaling, which is required for the stimulatory effect of diabetes on IL-6, markedly blunts the effect of diabetes on TNF-α and does not modulate diabetes-accelerated atherogenesis.

## Introduction

Both type 1 diabetes mellitus (T1DM) and type 2 diabetes mellitus (T2DM) are associated with macrovascular complications, which manifest as increased risks of myocardial infarction, stroke and peripheral vascular disease primarily due to increased atherosclerosis. The mechanisms whereby diabetes promotes atherosclerosis are incompletely understood. T1DM and T2DM are characterized by elevated blood glucose levels and are often associated with an increased inflammatory state, while other cardiovascular risk factors including dyslipidemia and insulin resistance are also present, in particularly in subjects with T2DM. Additional risk factors, such as hypertension, smoking, and nephropathy, when present, are also likely to play important roles in increasing cardiovascular disease risk, as they do in patients without diabetes. While tight control of glycemia has been demonstrated to reduce the risk of future cardiovascular events in young patients with T1DM [1, 2], the role of elevated glucose, lipids, inflammation and other factors associated with T1DM and cardiovascular disease risk are incompletely understood.

Myeloid cells isolated from diabetic humans and animal models often exhibit increased activation, resulting in increased expression of chemokines and cytokines, and Th17 cell expansion [3–8]. Furthermore, diabetes has been shown to result in increased inflammatory myelopoiesis in mouse models [9]. The inflammatory state of myeloid cells in diabetes might explain at least some of the effects of diabetes on atherosclerosis.

One of the possible mediators of increased inflammatory activation of myeloid cells in diabetes is prostaglandin E_2_ (PGE_2_). PGE_2_ stimulates expression of several inflammatory mediators and processes in myeloid cells, including IL-6 and the chemokine receptor CCR7 [10–12], while inhibiting others, e.g. TNF-α, CCL5 and inflammasome activation [11, 13, 14]. An explanation of PGE_2_’s divergent effects lies in the fact that there are four G protein–coupled PGE_2_ receptors (EP1-4), whereof EP4 has been most clearly linked to inflammation [15]. Furthermore, PGE_2_-mediated activation of EP4 induces several intracellular signaling events, including a rise in cyclic AMP (cAMP) and subsequent activation of the transcription factor CREB (cAMP-response element-binding protein), activation of phosphatidylinositol 3-kinase [16], and, at least in macrophages, inhibition of NF-κB after activation of toll-like receptor 4 with lipopolysaccharide [17]. PGE_2_ synthesis is stimulated by a large number of inflammatory mediators and, as a result, is often elevated in states of increased inflammation [18]. Thus, studies have shown increased plasma or urinary levels of PGE_2_ in patients with T1DM [19, 20], while others have found no differences [21]. Indeed, PGE_2_ has been suggested to mediate some of the complications associated with diabetes [20, 22–24]. For example, an EP4 agonist exacerbated renal fibrosis in streptozotocin-diabetic mice and enhanced diabetes-induced expression of inflammatory cytokines, including IL-6 [25]. The role of EP4 in macrovascular complications of diabetes is unknown, although a previous study from our laboratory implicated increased PGE_2_ release from macrophages in the inflammatory activation of these cells in a mouse model of T1DM [3].

To evaluate the role of EP4 in myeloid cells in diabetic and non-diabetic mice and in atherosclerosis associated with diabetes, we used the same [3] mouse model of T1DM, and transplanted these mice with bone marrow from newly generated myeloid cell-targeted EP4-deficient mice and littermate controls. Our results suggest that this mouse model of T1DM is associated with elevated myeloid cell PGE_2_-EP4 signaling, and that increased inflammatory activation of macrophages in diabetic mice, but not atherosclerosis, is dependent on myeloid cell EP4.

## Materials and Methods

### Generation of a mouse model of myeloid cell EP4-deficiency

Generation and genotyping of *Ptger4*-floxed mice have been described previously [26]. These mice were backcrossed 10 generations into the C57BL/6 background and then further crossed with *Lyz2-Cre*^*Tg*^ mice on the C57BL/6 background (B6.129P2-Lyz2^tm1(cre)Ifo^/J– 004781; Jackson Labs, Sacramento, CA) to generate myeloid cell-targeted EP4-deficient mice. *Lyz2-Cre* recombinase and WT fragments were detected using primer sequences provided by Jackson Labs (oIMR3066-3068). *Lyz2-Cre*^*Tg/Tg*^; *Ptger4*^*fl/fl*^ mice were used as myeloid cell-targeted EP4 –deficient (EP4^M-/-^) mice while *Lyz2-Cre*^*Tg/Tg*^; *Ptger4*^*wt/wt*^ littermates were used as wild type (WT) controls. Pilot studies demonstrated that macrophages from singly transgenic *Lyz2-Cre*^*Tg/wt*^; *Ptger4*^*fl/fl*^ mice did not exhibit significant loss of *Ptger4* mRNA. This study was carried out in strict accordance with the recommendations in the Guide for the Care and Use of Laboratory Animals of the National Institutes of Health. The protocol was approved by the Institutional Animal Care and Use Committee at the University of Washington (IACUC Protocol Number: 3154–01). All diabetic animals received insulin treatment as needed or were humanely euthanized by CO_2_ if they exhibited unexplained severe weight loss, lethargy, or symptoms indicative of severe illness/moribundity. All mice were euthanized by CO_2_ at the end of the experiment.

### Bone marrow transplants and induction of diabetes

The model of type 1 diabetes (*Ldlr*^*−/−*^;*Gp*^*Tg*^), in which diabetes can be induced at will using viral mimicry with lymphocytic choriomeningitis virus (LCMV), has been described previously [3, 27, 28]. Bone marrow transplants and induction of diabetes by LCMV were performed as previously described. Briefly, adult female *Ldlr*^*−/−*^;*Gp*^*Tg*^ mice 8–12 weeks of age [27] were lethally irradiated and the following day were injected with bone marrow from EP4^M-/-^ mice or littermate controls through the retro-orbital plexus. The mice were allowed to recover for 7–8 weeks before diabetes induction. Bone marrow transplanted mice were injected with LCMV (1 × 10^5^ pfu) or saline (control). One week after injection, at the onset of diabetes, the mice were switched from regular chow (PicoLab^®^ Rodent Diet 20, LabDiet, St. Louis, MO) to a low fat semipurified diet [27] and maintained for 12 weeks. The low fat semi-purified diet was used because when fed this diet, diabetic and non-diabetic mice have similar plasma cholesterol levels, which allows for analysis of the effect of diabetes *per se* on inflammation and atherogenesis, without marked dyslipidemia associated with diabetes, as described previously [27]. Dyslipidemia overrides the effects of diabetes on atherogenesis.

### Measurements of blood glucose, plasma lipids and white blood cell differentials

Blood glucose levels were determined by a stick test (OneTouch Ultra^®^, LifeScan Inc., Milpitas, CA), using blood from the saphenous vein, as described previously [27]. Plasma cholesterol levels were determined by the Cholesterol E kit (Wako Diagnostics, Wako, TX), and triglycerides were determined by a colorimetric kit from Wako Diagnostics [3]. EP4 has been reported to regulate bone marrow progenitor cells [29, 30], and blood levels of leukocyte populations were therefore determined as follows: Blood was collected from the retro-orbital plexus under isoflurane sedation. For total white blood cell (WBC) differentials, 30 μl blood was analyzed on a Hemavet (Drew Scientific, Miami Lakes, FL).

### In vitro myeloid cell experiments

Resident peritoneal macrophages were isolated as previously described [31]. After adhering to plates for 2–4 h, cells were washed three times with PBS, and were then maintained in DMEM (4.5 mmol/l glucose) with 10% fetal bovine serum and 100 pg/ml streptomycin sulfate and 100 units/ml penicillin G overnight. Generation of bone marrow-derived dendritic cells (BMDCs) and bone marrow-derived macrophages (BMDMs) was performed as described previously [32]. Bone marrow neutrophils were isolated on a 62% Percoll gradient. PGE_2_ (Cayman Chemical, Ann Arbor, MI) was used at a final concentration of 10 nmol/l. The toll-like receptor 4 ligand lipopolysaccharide (LPS) was obtained from Sigma (St. Louis, MO) and was used at a final concentration of 5 ng/ml.

### Real-time PCR, ELISAs and multiplex cytokine assays

Real-time PCR was performed as described by Kanter et al. [3]. RNA from cells was isolated using NucleoSpin^®^ RNA II Columns from Clontech (Mountain View, CA). RNA from tissues was isolated using RNeasy Fibrous Tissue Mini Kit (Valencia, CA). All reactions were treated with DNase to removed trace genomic DNA. The reverse-transcription reaction was carried out with ThermoFisher RevertAid Reverse Transcriptase kit (Waltham, MA). Real-time PCR products were confirmed by melting curve analysis. Quantitations were normalized to the *Rn18s* rRNA level in each reaction. Primers used for real-time PCR were as follows: *Il1b* forward GGGCTGCTTCCAAACCTTTG and reverse TGATACTGCCTGCCTGAAGCTC, *Ptger1* forward TGCTTGCCATCGACCTAGC and reverse CACCCAGGAAATGACACGC, *Ptger2* forward TCCCTAAAGGAAAAGTGGGACC and reverse GAGCGCATTAACCTCAGGACC, *Ptger3* forward CCGGAGCACTCTGCTGAAG and reverse CCCCACTAAGTCGGTGAGC, *Ptger4* forward ACCATTCCTAGATCGAACCGT and reverse CACCACCCCGAAGATGAACAT, *Il23a* forward AATAATGTGCCCCGTATCCAGT and reverse GCTCCCCTTTGAAGATGTCAG, and *Ccr7* forward TGTACGAGTCGGTGTGCTTC and reverse GGTAGGTATCCGTCATGGTCTTG. Other primer sequences have been published [33]. Mouse TNF-α and IL-6 ELISAs were obtained from eBioscience (San Diego, CA), and were used according to the manufacturer’s instructions. The PGE metabolite ELISA was obtained from Cayman Chemical (Ann Arbor, MI) and was used according to the manufacturer’s instructions. This ELISA detects the PGE metabolites 13,14-dihydro-15-keto PGA_2_ and 13,14-dihydro-15-keto PGE_2_. Luminex^®^ assays for plasma levels of IL-6 and TNF-α were obtained from R&D Systems, Inc. (Minneapolis, MN) and were performed according to the manufacturer’s instructions.

### Evaluation of atherogenesis

At the end of the study, mice were euthanized and blood was collected via heart puncture. The mice were perfused under physiological pressure and aortas were used for *en face* quantifications of atherosclerosis. The aortic sinus was serial sectioned (5 μm sections) and used for morphometric and immunohistological analyses, as described previously [27, 34]. In short, 68 aortic sinus sections/mouse were collected by serial sectioning in the proximal to distal direction, beginning at the level of attachment of the aortic valve cusps to the aorta and ending at the right and/or left coronary artery (a total of approximately 340 μm). Sections (two adjacent sections every 20 μm; 24 sections in total/mouse) were analyzed for the presence or absence of morphological features, and the results were expressed as frequencies/mouse and then expressed as mean ± SEM for each treatment group. All atherosclerosis analyses were performed by an observer blinded to the treatment groups.

### Statistical analysis

Statistical analysis was performed using two-tailed unpaired Student’s t-test, one-way ANOVA with Tukey’s multiple comparison, or two-way ANOVA followed by Tukey’s multiple comparison, as appropriate. For nonparametric analysis of sinus lesion morphology a Kruskal-Wallis test was used. Statistical outliers were identified by Grubbs’ test, as indicated in the figure legends. Probabilities <0.05 were considered statistically significant. *In vitro* experiments were performed at least three times in independent experiments.

## Results

### Diabetes is associated with increased plasma PGE metabolites

**Fig 1:**
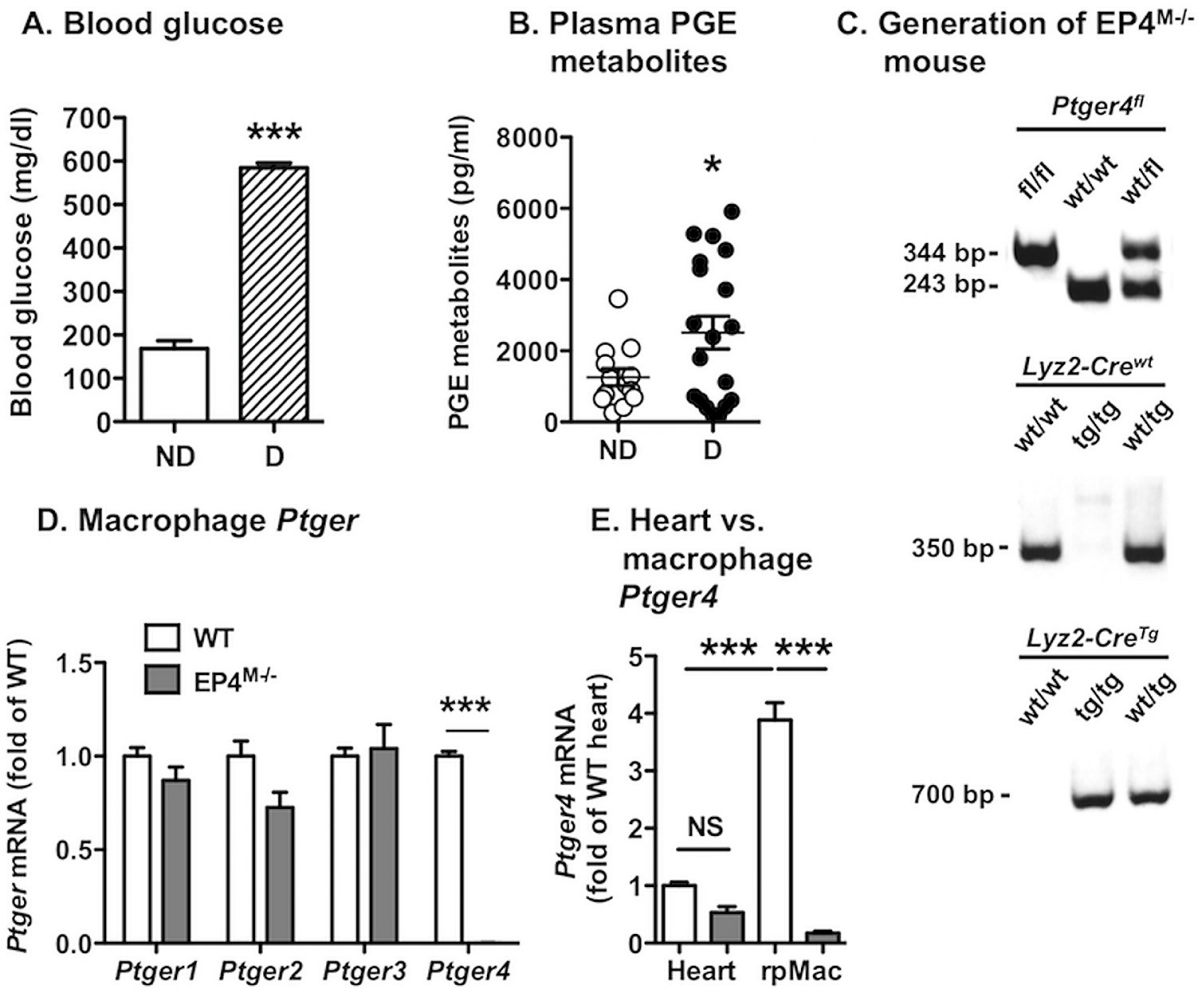
Rationale for generation of a myeloid cell-targeted EP4-deficient mouse model. Plasma and resident peritoneal macrophages were isolated from *Ldlr*^*-/-*^*; Gp*^*Tg*^ mice 12 weeks after induction of diabetes and from non-diabetic littermate controls. Glucose levels were measured in blood from the saphenous vein by a stick test (A). Plasma PGE metabolites were measured by ELISA (B). The pups from the *Ptger4*^*fl/fl*^ *x Lyz2-Cre*^*Tg/Tg*^ cross were genotyped as described in Materials and Methods (C). Resident peritoneal macrophages (rpMac) (D) and hearts (E) were harvested from EP4^M-/-^ mice and WT littermate controls, and *Ptger1-4* mRNA levels were measured by real-time PCR. The results are presented and mean ± SEM. Data in A-B (n = 9–22) were analyzed by unpaired two-tailed Student’s *t-*test and data in D-E were analyzed by one-way ANOVA followed by Tukey’s multiple comparison test (n = 5–7). Statistical outliers were identified by Grubbs’ test and were excluded from the analysis (one outlier in B), * p<0.05; *** p<0.001; NS, non-significant; ND, non-diabetic; D, diabetic.

Plasma PGE levels are increased in some studies of humans with T1DM [19, 20]. Because PGE_2_ is rapidly converted to metabolites in plasma, we measured plasma PGE_2_ metabolites in non-diabetic and diabetic *Ldlr*^*-/-*^*; Gp*^*Tg*^ mice. This virally-induced mouse model of T1DM has been described previously [3, 27]. Diabetic mice were hyperglycemic, as expected (Fig 1A). A subset of diabetic mice exhibited elevated plasma PGE metabolite levels, as compared with non-diabetic littermate controls, while others did not (Fig 1B). Together, the diabetic group had significantly higher PGE metabolite levels than non-diabetic littermates (Fig 1B). This mouse model of T1DM is therefore characterized by increased PGE status, consistent with our previous studies in which macrophages from diabetic mice were shown to secrete more PGE_2_ than macrophages from non-diabetic controls [3].

### The generated myeloid cell-targeted EP4-deficient (EP4^M-/-^) mouse exhibits a specific loss of *Ptger4* in myeloid cells

Because the PGE_2_ receptor EP4 (*Ptger4*) has been implicated in inflammatory activation associated with diabetes, we generated a mouse model of myeloid cell-targeted EP4-deficiency by taking advantage of the EP4-floxed mouse and *Lyz2-Cre*^*Tg*^ mice, which express Cre recombinase in myeloid cells. Following backcrossing for 10 generations into the C57BL/6 background, *Lyz2-Cre*^*Tg/Tg*^ *Ptger4*^*fl/fl*^ mice and *Lyz2-Cre*^*Tg/Tg*^ *Ptger4*^*wt/wt*^ littermate controls (Fig 1C) were used to measure *Ptger* mRNA levels in resident peritoneal macrophages and heart, to evaluate specificity of the EP4-deficiency. *Ptger4* mRNA was absent in macrophages from EP4^M-/-^ (*Lyz2-Cre*^*Tg/Tg*^ *Ptger4*^*fl/fl*^) mice, as compared with wildtype (WT; *Lyz2-Cre*^*Tg/Tg*^ *Ptger4*^*wt/wt*^) littermates (Fig 1D). There was no significant compensatory regulation of *Ptger1*, *Ptger2* or *Ptger3* mRNA. Furthermore, levels of *Ptger4* mRNA were not significantly reduced in hearts of EP4^M-/-^ mice (Fig 1E), demonstrating cell-specific *Ptger4* deletion.

### PGE_2_ exerts divergent effects on inflammatory mediators through EP4-dependent pathways in myeloid cells

**Fig 2:**
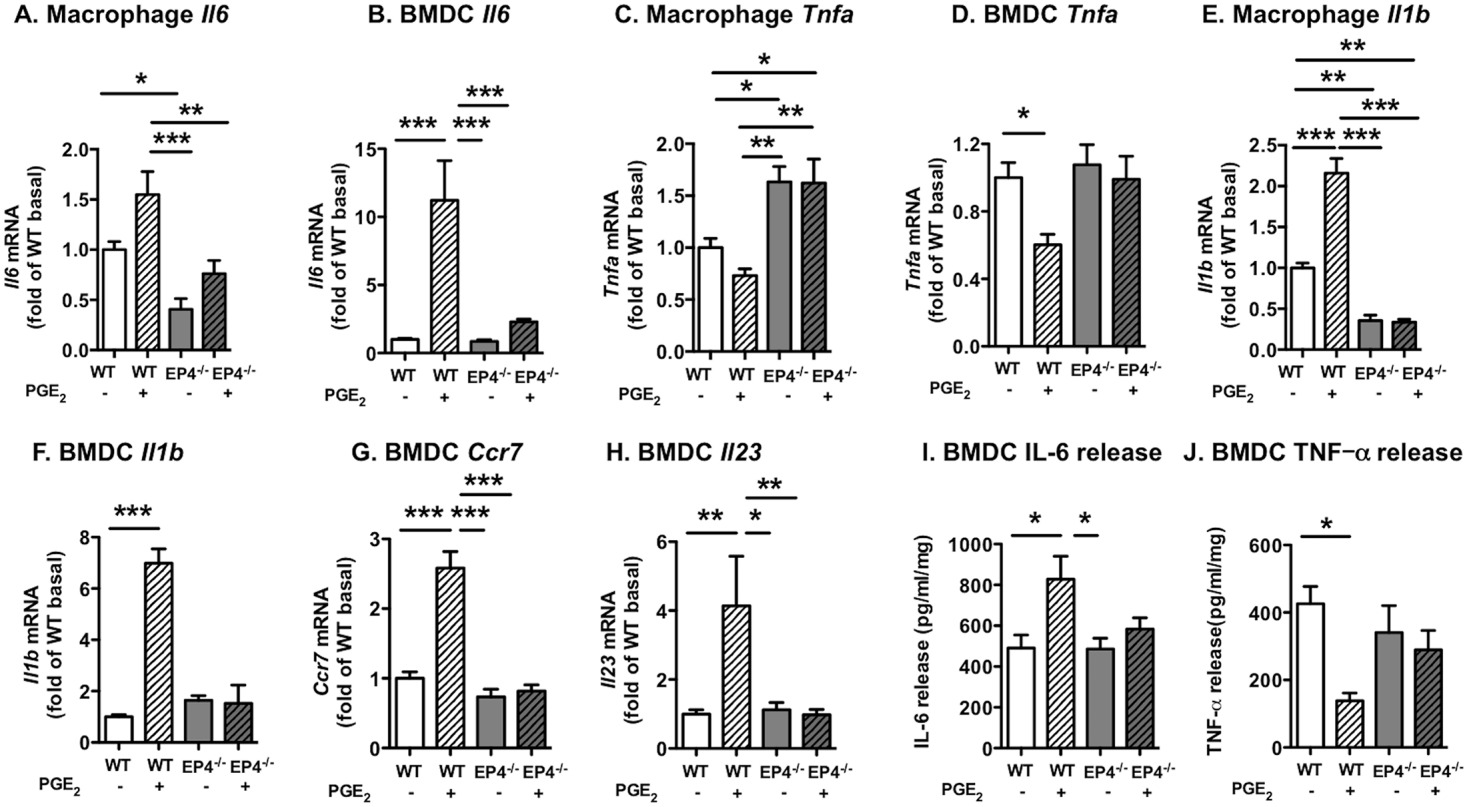
PGE_2_ exerts divergent effects on inflammatory mediators through EP4 in myeloid cells. Bone marrow-derived dendritic cells (BMDCs) and resident peritoneal macrophages from EP4^M-/-^ mice and WT littermates were stimulated with 10 nmol/l PGE_2_ or vehicle for 8 h. *Il6* mRNA (A-B), *Tnfa* mRNA (C-D), *Il1b* mRNA (E-F), *Ccr7* mRNA (G), and *Il23* mRNA (H) were measured by real-time PCR. IL-6 (I) and TNF-α (J) release was quantified by ELISA. The results are presented and mean ± SEM. Data were analyzed by two-way ANOVA with Tukey’s multiple comparisons test (real-time PCR data; 4–11; ELISA data; n = 4–6). Statistical outliers were identified by Grubbs’ test and were excluded from analyses (one outlier in A, two outliers in E), * p<0.05; ** p<0.01; *** p<0.001.

Having confirmed that the loss of EP4 is selective for myeloid cells and does not lead to compensatory changes in other PGE_2_ receptors under baseline conditions, we explored the role of EP4 in mediating effects of PGE_2_ on several inflammatory mediators in four types of myeloid cells; resident peritoneal macrophages, bone marrow-derived dendritic cells (BMDCs), bone marrow-derived macrophages (BMDMs), and bone marrow neutrophils. *Ptger4* was poorly expressed in neutrophils, and these cells were therefore not studied further. PGE_2_ (10 nmol/l) stimulated *Il6* mRNA levels in both isolated peritoneal resident macrophages and BMDCs at 8 h and this effect was largely mediated by EP4 (Fig 2A and 2B). Conversely, the same concentration of PGE_2_ inhibited *Tnfa* mRNA levels through EP4 (Fig 2C and 2D). The fact that EP4-deficiency increased macrophage *Tnfa* to levels higher than those in WT macrophages in the absence of PGE_2_ suggests that endogenous PGE_2_ secretion was elevated in peritoneal macrophages, as compared with BMDCs.

PGE_2_ also increased levels of *Il1b* and *Il23* as well as *Ccr7*, a chemokine receptor that mediates migration of dendritic cells to lymph nodes [35] through EP4-dependent mechanisms (Fig 2E–2H). The ability of PGE_2_ to stimulate IL-6 and inhibit TNF-α in BMDCs was replicated at the protein level by ELISAs (Fig 2I and 2J). Furthermore, PGE_2_ inhibited *Tnfa* mRNA levels also in BMDMs through EP4 (wildtype basal *Tnfa* 0.97 ± 0.03; wildtype PGE_2_ 0.46 ± 0.02; p<0.001 versus wildtype basal; EP4^M-/-^ basal 1.08 ± 0.09; EP4^M-/-^ PGE_2_ 0.90 ± 0.10; mean ± SEM; n = 9–11), confirming this effect of PGE_2_ in second macrophage population. Levels of *Il6* mRNA were not detected or were very low in the basal state or after PGE_2_ stimulation of BMDMs. We therefore focused our studies primarily on resident peritoneal macrophages, rather than BMDMs. An additional reason to study resident peritoneal macrophages was that the effect of diabetes on resident peritoneal macrophages could be assessed, as discussed below.

Our findings suggest that the low concentration of PGE_2_ used (10 nmol/l) mediates its effects primarily through EP4. However, we observed EP4-independent effects of a higher concentration of PGE_2_ (50 nmol/l). Indeed, 10 nmol/l PGE_2_ has been shown to increase IL-23 production in human monocyte-derived dendritic cells through EP4, similar to the results of the present study, but to downregulate IL-23 at higher concentrations (>50 nmol/l) through interaction with EP2 [36].

### PGE_2_ prevents inflammatory effects of LPS through EP4 in myeloid cells

**Fig 3:**
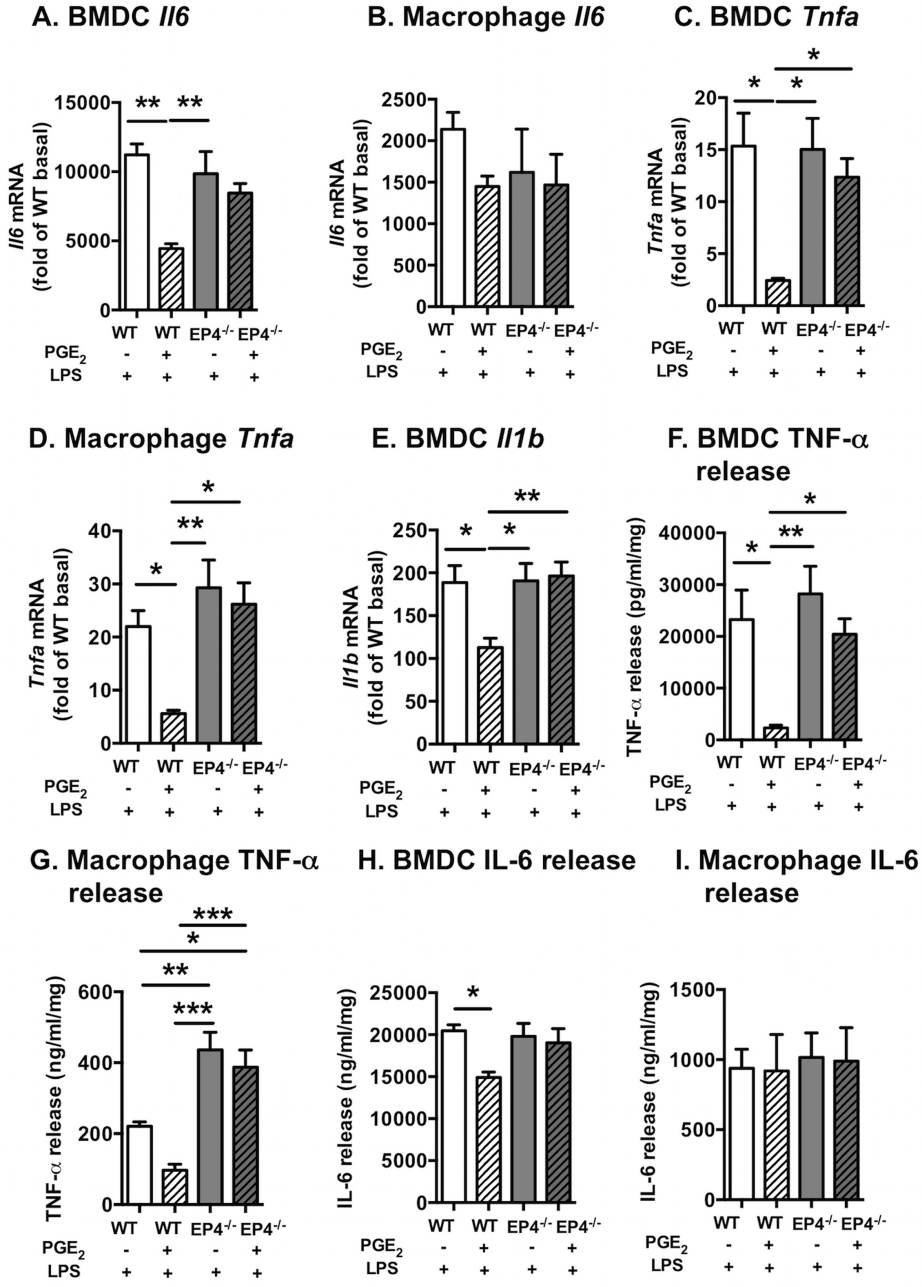
PGE_2_ inhibits LPS-induced cytokines through EP4 in myeloid cells. Bone marrow-derived dendritic cells (BMDCs) and resident peritoneal macrophages from EP4^M-/-^ mice and WT littermates were stimulated with 10 nmol/l PGE_2_ or vehicle for 2 h, and then for an additional 6 h in the presence or absence of 5 ng/ml LPS. *Il6* mRNA (A-B), *Tnfa* mRNA (C-D), and *Il1b* mRNA (E) were measured by real-time PCR. TNF-α (F-G) and IL-6 (H-I) release was quantified by ELISA. The results are presented as fold over WT cells incubated in the absence of LPS as mean ± SEM. Data were analyzed by two-way ANOVA with Tukey's multiple comparisons test (n = 9–11). * p<0.05; ** p<0.01; *** p<0.001.

It is well-established that PGE_2_ signaling through EP4 suppresses the effects of LPS on cytokine production in macrophages by preventing LPS-mediated NF-κB activation [17, 37, 38]. The mechanism of PGE_2_-EP4-mediated inhibition of cytokine production in macrophages involves the protein EPRAP [prostaglandin E receptor (EP) 4-associated protein; gene name *Fem1a*], which acts through a non-cyclic AMP-dependent pathway to inhibit macrophage activation through direct interaction and stabilization of NF-κB1 p50/p105 following exposure to a pro-inflammatory stimulus, such as LPS [17, 39]. We therefore next investigated the effects of PGE_2_-EP4 on LPS-induced cytokine production in BMDCs and resident macrophages to further characterize our myeloid cell-targeted EP4-deficient model and to verify previous results. Contrary to the stimulatory effects of PGE_2_ on IL-6 production in the absence of LPS (Fig 2), PGE_2_ suppressed the effects of LPS on *Il6* mRNA in BMDCs (Fig 3A). This effect was not seen in resident macrophages (Fig 3B). PGE_2_ also markedly suppressed LPS-induced *Tnfa and Il1b* levels in both BMDCs and resident macrophages (Fig 3C–3E), and this effect was dependent on EP4, consistent with previous studies by the Libby laboratory [17]. The suppressive effects of PGE_2_ on LPS-induced IL-6 and TNF-α in BMDCs were confirmed at the protein level by ELISAs (Fig 3F and 3H), although the effect of PGE_2_ was more marked for TNF-α. Similarly, EP4 suppressed LPS-mediated TNF-α in resident peritoneal macrophages, while IL-6 release was unaffected (Fig 3G and 3I). Together, these results show that whereas PGE_2_ alone promotes production of several inflammatory mediators, it suppresses the action of LPS, consistent with previous studies [17, 37]. Both actions of PGE_2_ are critically dependent on EP4.

**Fig 4:**
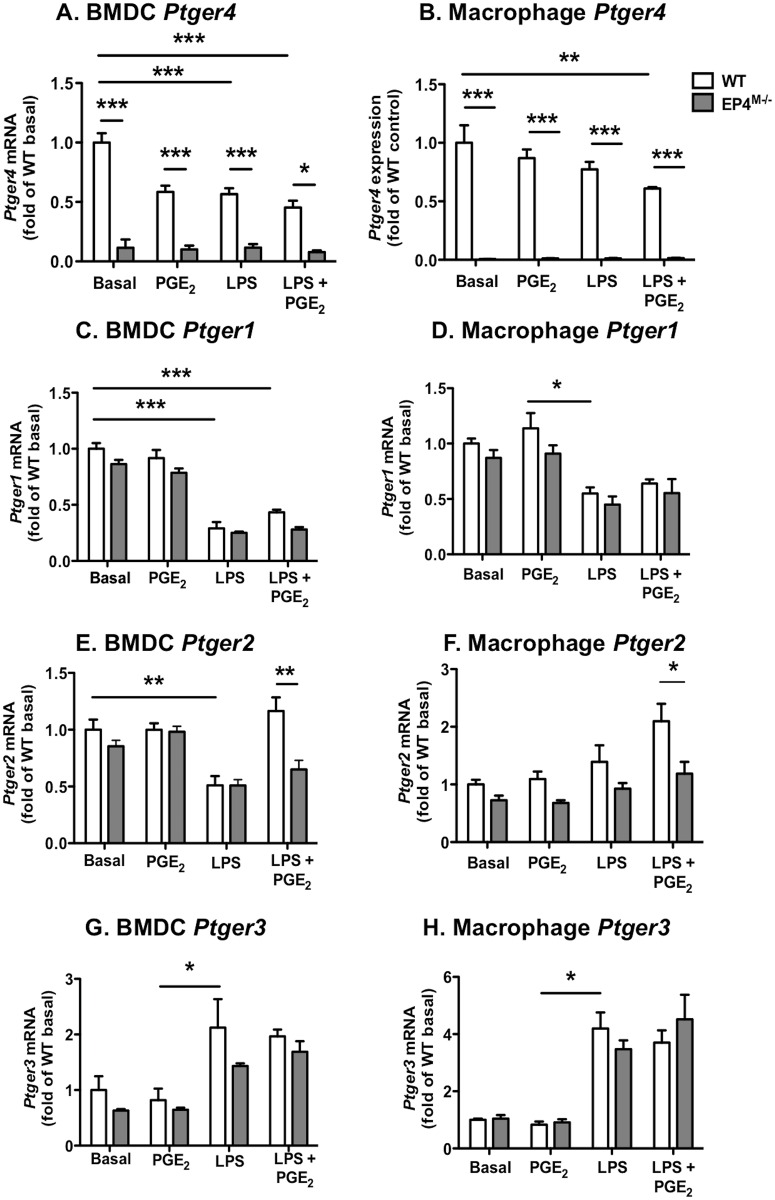
PGE_2_ receptors are differentially regulated by LPS and PGE_2_ in myeloid cells. Bone marrow-derived dendritic cells (BMDCs) and resident peritoneal macrophages from EP4^M-/-^ mice and WT littermates were stimulated with 10 nmol/l PGE_2_ or vehicle for 2 h, and then for an additional 6 h in the presence or absence of 5 ng/ml LPS. *Ptger4* mRNA (A-B), *Ptger1* mRNA (C-D), *Ptger2* mRNA (E-F) and *Ptger3* mRNA (G-H) were measured by real-time PCR. The results are presented and mean ± SEM. Data were analyzed by one-way ANOVA with Tukey's multiple comparisons test (n = 7–11). * p<0.05; ** p<0.01; *** p<0.001.

We next investigated regulation of the four PGE_2_ receptors (*Ptger1-4*) in both BMDCs and resident peritoneal macrophages from EP4^M-/-^ mice and WT littermate controls in response to PGE_2_ and LPS, to further evaluate the possibility of compensatory regulation of EP1, EP2 and EP3. Levels of *Ptger4* and *Ptger1* mRNA were modestly reduced by LPS stimulation in both BMDCs and resident peritoneal macrophages (Fig 4A–4D). Likewise, *Ptger2* mRNA was suppressed by LPS in BMDCs, but this effect was not consistent with that on macrophages (Fig 4E and 4F). Interestingly, EP4-deficiency resulted in a significant downregulation of *Ptger2* mRNA in both BMDCs and resident macrophages stimulated with a combination of LPS and PGE_2_ (Fig 4E and 4F). These results suggest that the expression of EP2 is dependent on EP4 expression under certain conditions. Finally, *Ptger3* mRNA levels were significantly increased in LPS-stimulated BMDCs and resident macrophages (Fig 4G and 4H). Together, these data demonstrate that the four PGE_2_ receptors exhibit different regulation in myeloid cells, and that the effect of EP4-deficiency in cells stimulated by PGE_2_ in the presence of LPS might be due in part to downregulation of EP2, but that in the absence of LPS, there is no major compensatory regulation of other PGE_2_ receptors by EP4-deficiency.

### Myeloid cell-targeted EP4-deficiency does not alter diabetes induction, plasma lipid levels or white blood cell counts

**Fig 5:**
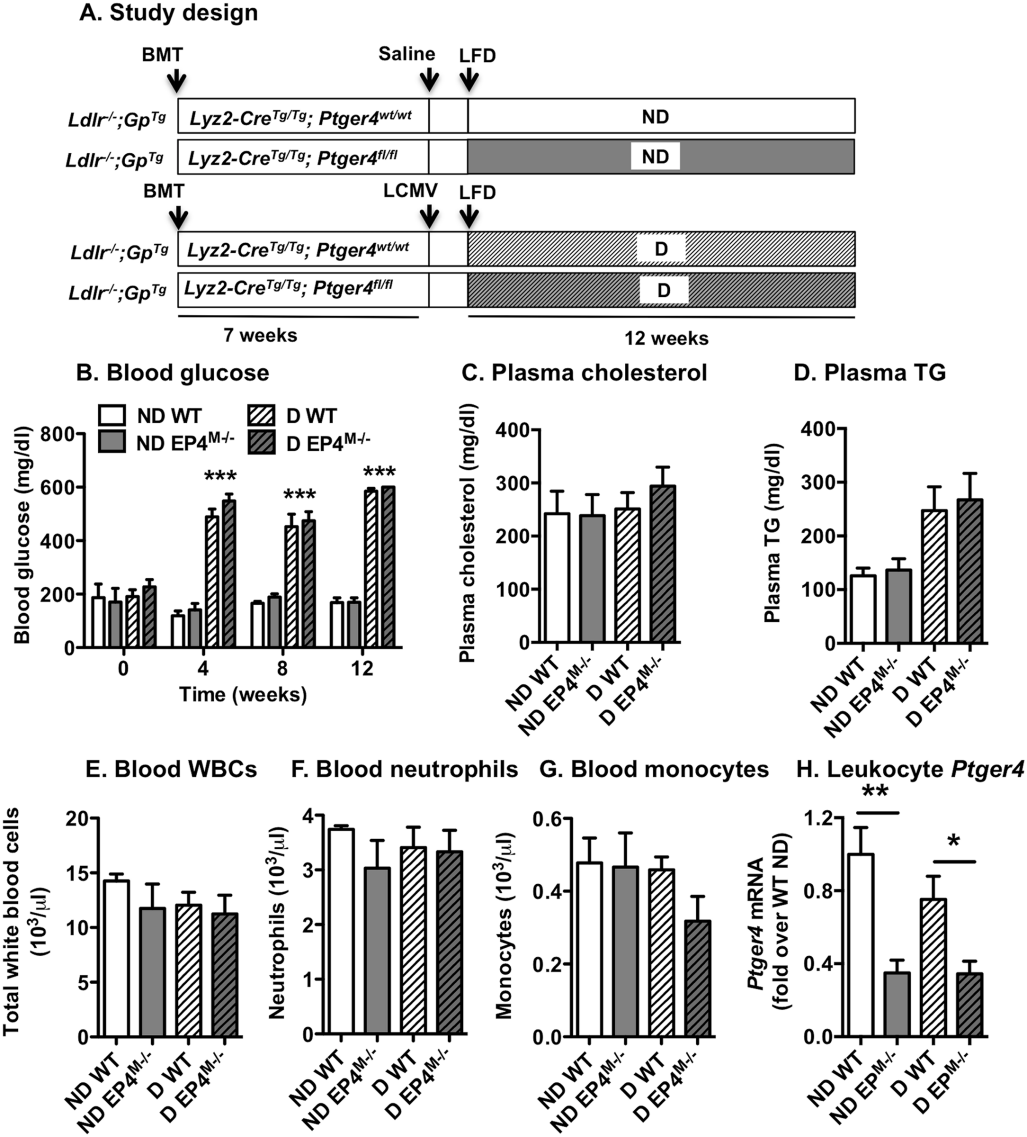
Myeloid cell EP4-deficiency does not alter diabetes induction, plasma lipid levels or WBC counts. The study plan in shown in A. Blood glucose levels were measured at week 0 (prior to injection of LCMV), 4, 8 and 12 by a stick test (B). Plasma cholesterol (C) and triglycerides (D) were measured by kits from Wako. Blood leukocyte counts were determined by a Hemavet (E-G). Leukocyte *Ptger4* mRNA levels were measured by real-time PCR (H). The results are presented and mean ± SEM. Data were analyzed by one-way ANOVA with Tukey's multiple comparisons test (n = 5–11 in B-C; n = 9–14 in D; 4–7 in E-G and 14–21 in H). * p<0.05; ** p<0.01; *** p<0.001; ND, non-diabetic; D, diabetic; LCMV, lymphocytic choriomeningitis virus; LFD, low-fat diet.

Hematopoietic EP4-deficiency has been shown to prevent lesions of atherosclerosis [40] or to have no impact on lesion size [41] in non-diabetic fat-fed *Ldlr*^*-/-*^ mice. We reasoned that the mouse model of T1DM might be more susceptible to differences in myeloid cell EP4-deficiency because plasma PGE metabolites were elevated. Furthermore, the two previous studies were performed in mice that lacked EP4 in all bone marrow-derived cells, whereas our model of EP4-deficiency targeted to myeloid cells is the first to investigate effects on atherosclerosis. The study plan is shown in Fig 5A. EP4^M-/-^ mice and WT littermate controls were used as donors for bone marrow transplants into female *Ldlr*^*-/-*^*; Gp*^*Tg*^ mice (the model of T1DM). The mice were allowed to recover for 7 weeks following the bone marrow transplant, and were then injected with LCMV to induce diabetes or saline as control (Fig 5A). All mice were fed a low-fat semi-purified diet, described previously [27], for an additional 12 weeks after induction of diabetes.

Diabetic mice were hyperglycemic at 4 weeks after induction of diabetes, and maintained hyperglycemia throughout the study (Fig 5B). Myeloid cell EP4-deficiency did not alter hyperglycemia or diabetes induction in this model of diabetes, consistent with data on global EP4-deficiency in the streptozotocin-induced diabetes model [42]. Plasma cholesterol levels and triglyceride levels were not significantly different in the four groups of mice (Fig 5C and 5D), although triglyceride levels tended to be increased in diabetic mice. Because EP4 has been shown to regulate bone marrow progenitor cells, we next evaluated numbers of blood leukocytes. There were no significant differences between the groups in total leukocytes, neutrophils, monocytes (Fig 5E–5G) or lymphocytes (ND WT 9.6 ± 0.5 x 10^3^ cells/μl blood; ND EP4^M-/-^ 8.0 ± 1.6; D WT 8.0 ± 1.1 and D EP4^M-/-^ 7.5 ± 1.4 x 10^3^ cells/μl; mean ± SEM; n = 5–7). Plasma levels of IL-6 and TNF-α were below the detection limit of the assay (18.6 pg/ml for IL-6 and 1.1 pg/ml for TNF-α). Leukocyte mRNA levels of *Ptger4* were significantly reduced in both non-diabetic and diabetic mice that had received EP4^M-/-^ bone marrow, as compared with mice that received WT bone marrow transplants (Fig 5H). Thus, myeloid cell-targeted EP4-deficiency did not affect diabetes severity, plasma lipids or leukocyte numbers.

### Myeloid cell-targeted EP4-deficiency markedly modulates the effect of diabetes on mediators of inflammation in resident peritoneal macrophages

**Fig 6:**
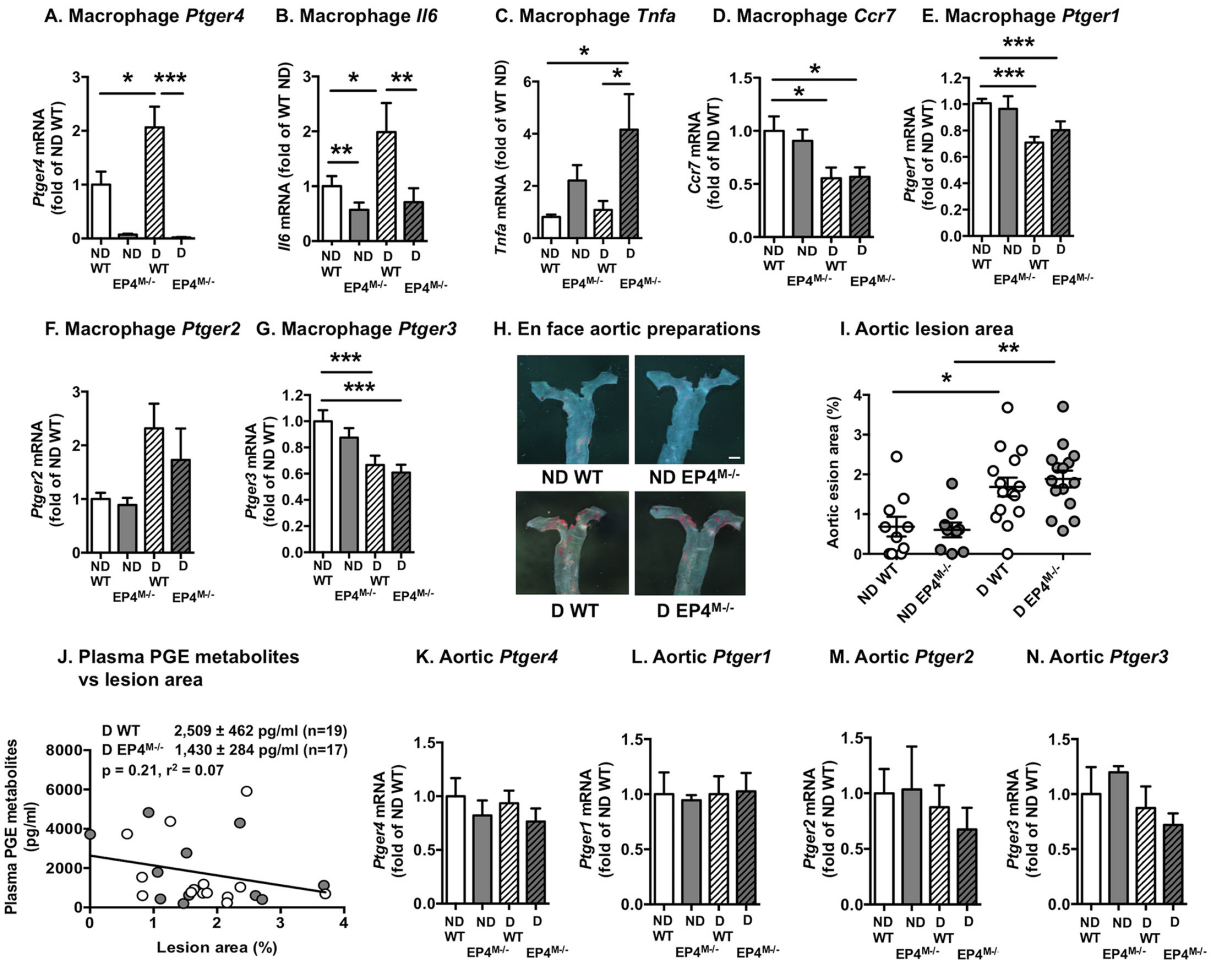
Myeloid cell EP4-deficiency modulates macrophage cytokines in diabetic mice, but does not impact aortic atherosclerosis. At the end of the study, resident peritoneal macrophages were collected from the four groups of mice, and mRNA levels of *Ptger4* (A), *Il6* (B), *Tnfa* (C), *Ccr7* (D), *Ptger1* (E), *Ptger2* (F), and *Ptger3* (G) were determined by real-time PCR. Aortic lesion area was measured on *en face* preparations stained with Sudan IV (H-I). Images of the thoracic aortas are shown in H. The results are presented and mean ± SEM. Data were analyzed by two-way ANOVA with Tukey's multiple comparisons test in A-G, I (n = 11–22 in A-D; 9–15 in I). Statistical outliers were identified by Grubbs’ test and excluded from data analysis (one outlier in D and E, two outliers in B and F, and three outliers in C), * p<0.05; ** p<0.01; *** p<0.00. Correlation between plasma PGE metabolite levels and aortic lesion area in diabetic mice was evaluated by Spearman correlation (J). The analysis included 25 mice; 12 wildtype mice and 13 EP4^M-/-^ mice. White symbols, WT mice; gray symbols EP4^M-/-^ mice. Aortic mRNA levels of *Ptger1-4* were measured in 4–6 mice/group at the end of the experiment (K-N).

After 12 weeks of diabetes, resident peritoneal macrophages were harvested from the four groups of mice. Both non-diabetic and diabetic mice that had received EP4^M-/-^ bone marrow demonstrated an almost complete lack of *Ptger4* mRNA in peritoneal macrophages, as compared with mice that had received WT bone marrow, indicating a near-complete chimerism (Fig 6A). *Ptger4* mRNA levels were higher in macrophages from wildtype diabetic mice, as compared with wildtype non-diabetic mice (Fig 6A). *Il6* mRNA levels were significantly higher in macrophages from diabetic mice that had received WT bone marrow, as compared with non-diabetic mice and diabetic mice that had received myeloid cell EP4-deficient bone marrow (Fig 6B), consistent with the ability of PGE_2_ to increase IL-6 through EP4 (Fig 2). Furthermore, diabetic mice that had received myeloid cell EP4-deficient bone marrow exhibited significantly higher levels of *Tnfa* mRNA than both non-diabetic WT mice and diabetic WT mice (Fig 6C). These results are also consistent with the ability of PGE_2_ to suppress TNF-α through EP4 (Fig 2). Myeloid cell EP4-deficiency had no statistically significant effect on *Tnfa* mRNA levels in non-diabetic mice. Thus, PGE_2_-EP4 has similar effects *in vitro* and *in vivo* on IL-6 and TNF-α in diabetic mice, and the effects of diabetes on *Il6* and *Tnfa* are dependent on myeloid cell EP4.

Conversely, diabetes resulted in suppression of *Ccr7* mRNA levels in macrophages through a non-EP4-dependent mechanism (Fig 6D). The effect of diabetes on *Ccr7* is consistent with a previous study showing reduced *Ccr7* mRNA levels in lesional macrophages from regressing lesions in diabetic mice [43]. These findings suggest that myeloid cell EP4 significantly impacts some inflammatory effects of diabetes, but not others.

Interestingly, diabetes resulted in a significant reduction of *Ptger1* (Fig 6E) and *Ptger3* mRNA (Fig 6G) levels in macrophages; effects that were not mediated by myeloid cell EP4. *Ptger2* mRNA levels tended to be increased in macrophages from diabetic mice, as compared with macrophages from non-diabetic mice, but this effect was not significant by ANOVA (Fig 6F). There were no significant effects of EP4-deficiency on *Ptger1-3* mRNA levels (Fig 6E–6G), suggesting that EP4-deficiency does not lead to compensatory effects on other macrophage PGE_2_ receptors *in vivo*.

### Myeloid cell-targeted EP4-deficiency does not impact atherogenesis or lesional macrophage accumulation in non-diabetic or diabetic mice

Finally, we evaluated atherosclerosis at two different sites; the full-length aorta and the aortic sinus. Aortic lesions were small, and diabetes caused increased aortic atherosclerosis, as we have demonstrated previously in this model [27, 28, 44]. This effect of diabetes was independent of myeloid cell EP4 expression (Fig 6H and 6I). Representative *en face* aortic preparations are shown in Fig 6H. Furthermore, there was no significant (p = 0.21) correlation between lesion area and plasma PGE metabolites in diabetic mice (Fig 6J), supporting the conclusion that increased PGE_2_ production does not explain diabetes-accelerated atherogenesis. Aortic levels of *Ptger1-4* were not significantly altered by diabetes or myeloid cell EP4-deficiency (Fig 6K–6N), indicating that neither diabetes nor myeloid cell EP4 affect smooth muscle cell expression of PGE_2_ receptors.

**Fig 7:**
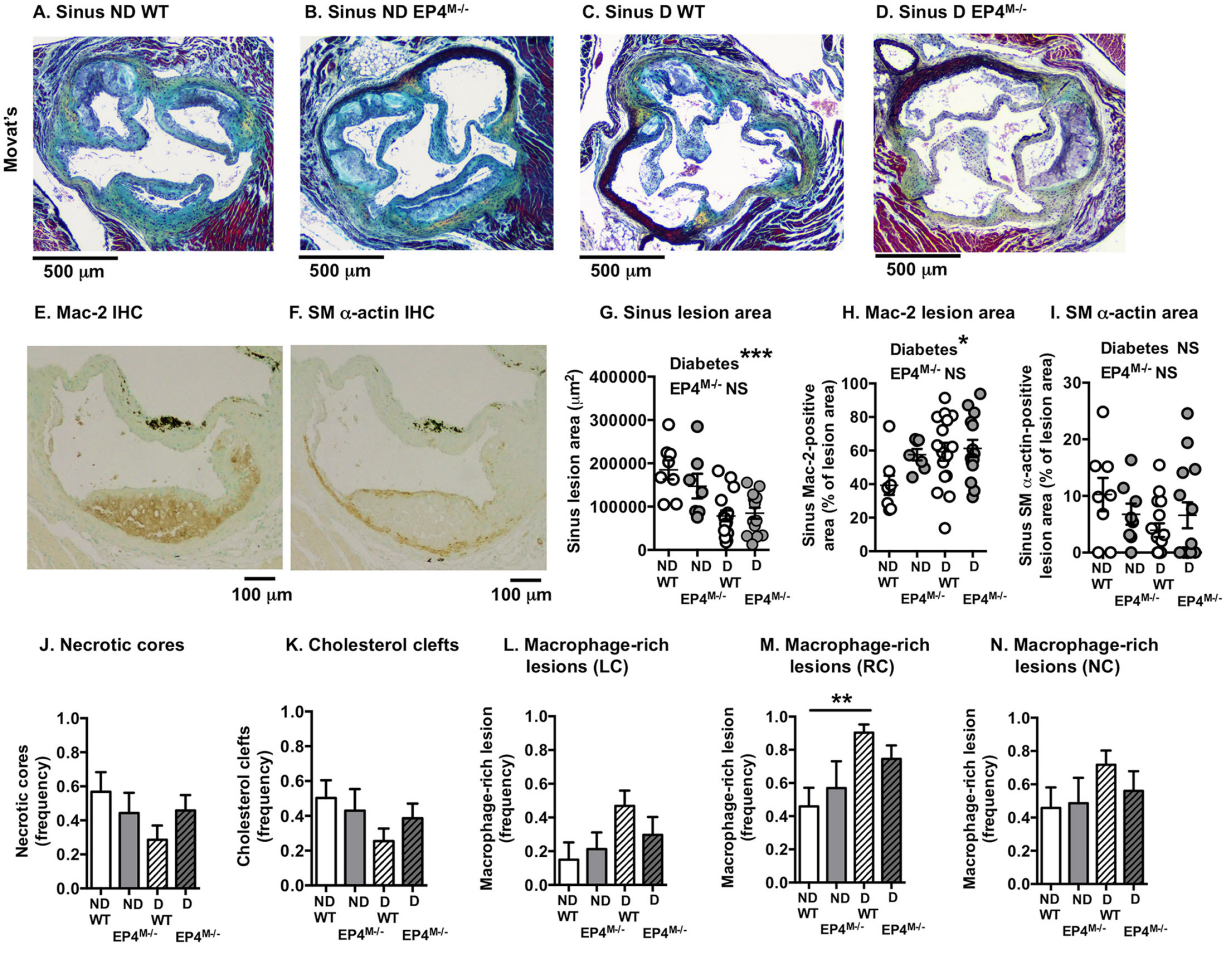
Myeloid cell EP4-deficiency does not impact atherosclerotic lesions in the aortic sinus. Representative aortic sinus lesion cross-sections stained with a Movat’s pentachrome stain from the four groups of mice are shown (A-D). Adjacent sections were immunostained for macrophages (using an anti-Mac-2 antibody; E) and smooth muscle cells (using a smooth muscle α-actin antibody; F). Cross-sectional lesion area (G), Mac-2-positive lesion area (H) and smooth muscle-positive lesion area (I) was quantified. Statistical analysis was performed by two-way ANOVA. (J-N) Twenty-four cross-sections/mouse were scored for presence or absence of left coronary sinus lesional necrotic cores and cholesterol clefts (J-K). Frequency of macrophage-rich lesions were scored for the three sinus lesions; left coronary (LC), right coronary (RC) and non-coronary (NC) (L-N). The results are presented and mean ± SEM. Data were analyzed by Kruskal-Wallis test (n = 8–15). There were no significant differences between the four groups. ND, non-diabetic; D, diabetic.

Aortic sinus lesions were used to evaluate effects of EP4-deficiency on lesion morphology. Overall, lesions were large and complex at this site (Fig 7A–7D). First, the effect of diabetes and myeloid cell EP4-deficiency on lesional macrophage and smooth muscle cell content was analyzed by immunohistochemistry (Fig 7E and 7F). Diabetes resulted in an overall reduction in sinus lesion area in both wildtype and EP4^M-/-^ mice (Fig 7G). However, the relative lesional area occupied by macrophages was increased in both groups of diabetic mice, with no significant effect on lesional smooth muscle cell content (Fig 7H and 7I). This appears to be consistent with previous studies showing that diabetes promotes lesional macrophage accumulation and monocyte recruitment into lesions [9, 27, 28]. Next, the left coronary sinus lesion was analyzed because this site contained the most advanced lesions and showed frequent necrotic cores in both non-diabetic and diabetic mice, and because lesional macrophage apoptosis has been shown to be increased by hematopoietic EP4-deficiency in a previous study of fat-fed mice [40]. The left coronary sinus lesions exhibited several traits of advanced lesions, such as necrotic cores, cholesterol clefts, fibrous caps and fibrous cap collagen. Neither diabetes nor myeloid cell EP4-deficiency had a significant effect on these hallmarks of advanced lesions (Fig 7J and 7K). Consistent with the effect of diabetes on macrophage accumulation, macrophage-rich lesions were more frequent in diabetic mice, especially in the right coronary sinus (Fig 7L and 7M), while myeloid cell EP4-deficiency had no effect.

## Discussion

This study demonstrates that PGE_2_ has divergent effects on myeloid cell cytokine production through EP4 in that it stimulates production of some cytokines (e.g. IL-6, IL-1β, and IL-23) while inhibiting production of others (e.g. TNF-α). This divergent effect of PGE_2_ is most likely due to the different signaling pathways activated following PGE_2_ binding to EP4 [16, 45]. The cAMP surge has been shown to induce a multitude of chemokines and cytokines in macrophages in the absence of LPS [46]. For example, it has been shown that PGE_2_ induces IL-6 through cAMP and a subsequent activation of CREB in fibroblasts [47], but that it suppresses TNF-α transcription through induction of Early Growth Response Factor-1 (Egr-1), which in turn results in suppression of cytokine-induced c-Jun in synovial fibroblasts and THP-1 cells [48]. PGE_2_-mediated induction of Egr-1 has been shown to occur through activation of phosphatidylinositol 3-kinase and extracellular signal-regulated kinases through EP4 [2]. The well-studied ability of EP4 to suppress NF-κB activation in macrophages [17] is also likely to contribute to the reduced TNF-α expression after PGE_2_ stimulation, and the ability of PGE_2_ to suppress IL-6 in LPS-stimulated macrophages. The ability of EP4 activation to suppress NF-κB signaling occurs, at least in part, through the protein EPRAP, which interacts directly with NF-κB1 p105/p50 and EP4 [17]. Thus, our study demonstrates that myeloid cell PGE_2_-EP4 signaling exerts divergent effects on cytokines likely depending on the signaling pathways associated with production of a given cytokine and the inflammatory milieu in which the cell resides.

We have previously shown that diabetes is associated with increased production of PGE_2_, IL-6 and TNF-α in macrophages [3]. The present study highlights the importance of EP4 in inflammatory activation induced by diabetes. Thus, EP4 was required for the effects of diabetes on *Il6* and markedly suppressed the effects of diabetes on *Tnfa* levels in macrophages. These results strongly suggest that diabetes promotes IL-6 production in myeloid cells through increased PGE_2_-EP4 signaling, whereas PGE_2_-EP4 signaling acts to suppress TNF-α production in the setting of diabetes. It is possible that these effects of diabetes are mediated by stimulation of TLR4 by endogenous ligands, and that there is cross-talk between TLR4 signaling and EP4 signaling in diabetes. Furthermore, we show that diabetes-accelerated atherogenesis is not dependent on PGE_2_-EP4 signaling in myeloid cells. Together, these results are important because PGE_2_ production is elevated in inflammatory states, including in some cases diabetes, and PGE_2_-EP4 has been shown to mediate detrimental effects on the kidney in diabetic mice through increased IL-6 production [22, 25]. Similarly, hematopoietic EP4-deficiency reduces inflammation in a mouse model of multiple sclerosis through a mechanism likely involving reduced IL-6 production [49], and EP4 is required for initiation of skin immune responses after antigen exposure by promoting migration of skin dendritic cells [50].

Conversely, hematopoietic EP4-deficiency has been shown to augment inflammation in other states; for example, hematopoietic EP4-deficiency enhances inflammation and aortic aneurysm formation in an *Ldlr*^*-/-*^ mouse model [51]. The role of PGE_2_ in modulating inflammatory processed and atherosclerosis is complex [44], and likely depends on the disease model, timing and cell types involved. The present study is the first, to the best of our knowledge, to address the role of myeloid cell EP4 in a mouse model of diabetes-accelerated atherogenesis. Our results indicate that diabetes acts through other mechanisms to promote atherosclerosis, and further suggest that macrophage production of IL-6 or TNF-α might not explain diabetes-accelerated atherogenesis since these cytokines were significantly altered by EP4-deficiency. However, we cannot rule out the possibility that the divergent effects of EP4 on IL-6 and TNF-α result in a zero sum effect on atherogenesis.

We show that *Ptger4* mRNA levels are elevated in resident peritoneal macrophages from diabetic mice, as compared with non-diabetic littermates. EP4 has previously been shown to be upregulated in macrophages from pristane-treated mice, a model of some aspects of lupus [11] and in a macrophage cell line by a combination of LPS and IFN-γ stimulation [52]. Our results also suggest that diabetes results in downregulation of EP1 and EP3 in macrophages. Since EP4 acts to increase cAMP levels and EP1 and EP3 act to reduce cAMP levels, the net effect is likely to be an increased cAMP load in macrophages subjected to PGE_2_ stimulation under diabetic conditions. Different cell types appear to respond differently to diabetes because EP3 is upregulated in islets from diabetic mice, as compared with controls [53].

Several recent papers have addressed the role of PGE_2_ in atherogenesis in different mouse models. Deletion of mPGES-1, its most proximal synthase, both globally and specifically in myeloid cells markedly reduces atherogenesis in hyperlipidemic *Ldlr*^*-/-*^ mice [54, 55]. Loss of hematopoietic EP2 was demonstrated to have no effect on atherogenesis in fat-fed *Ldlr*^*-/-*^ mice [40], while studies on EP4 contributions have produced contradictory results [40, 41]. In one study, loss of hematopoietic cell EP4 resulted in a reduction in early lesions, which was attributed to increased apoptosis of macrophages [40]. The EP4-deficient mice did not show differences in plasma lipoproteins, consistent with the present study, and thioglycollate-elicited EP4-deficient macrophages exhibited reduced levels of cytokines, including *Il6* [40]. These results are also consistent with our data on resident peritoneal macrophages, which showed a significant reduction in *Il6* by EP4-deficiency in diabetic mice. However, we also observed increased *Tnfa* in EP4-deficient macrophages from diabetic mice, demonstrating the complexity of PGE_2_’s effects on inflammatory pathways. In the present study, myeloid cell EP4-deficiency did not result in increased necrotic core formation in lesions, suggesting that macrophage apoptosis was not affected in this model of T1DM-accelerated atherosclerosis. The second study used a similar method to induce hematopoietic EP4-deficiency in fat-fed *Ldlr*^*-/-*^ mice [41]. No differences in lesion size were observed at 5 or 10 weeks after initiation of fat-feeding, consistent with the lack of effects of EP4-deficiency on atherosclerosis in our study. However, hematopoietic EP4-deficiency resulted in increased lesional macrophages and T cells, with no effect on apoptosis in lesional macrophages [41]. It is possible that the differences in atherosclerosis observed in fat-fed *Ldlr*^*-/-*^ mice with hematopoietic EP4-deficiency, as compared with our study, was due to the fat-feeding or that the inhibition of atherosclerosis was mediated by hematopoietic cells other than myeloid cells. The present study clearly demonstrates that myeloid cell-targeted EP4-deficiency alters cytokine production but has no effect on atherosclerosis in non-diabetic or diabetic mice. It is however possible that a greater effect would have been observed if both EP4 and EP2 had been deleted in myeloid cells.

Since myeloid cell EP4 expression does not impact diabetes-accelerated atherogenesis and there was no correlation between plasma PGE metabolites and lesion area in diabetic mice, what then is the mechanism whereby diabetes promotes atherosclerotic lesion initiation? The present study and published studies offer some insights into this question. For example, our study demonstrates that the increased atherogenesis in diabetic mice was not due to elevated cholesterol levels, as compared to non-diabetic mice, consistent with previous studies [3, 27]. Furthermore, we did not observe myelopoiesis and neutrophilia in diabetic mice in this study, suggesting that diabetes-accelerated lesion initiation was not due to elevated levels of circulating myeloid cells. Moreover, we have recently shown that increased glucose flux in myeloid cells is not sufficient to stimulate atherosclerosis in *Ldlr*^*-/-*^ mice [33], but it is quite possible that hyperglycemia plays a role in increasing myeloid cell accumulation in lesions through other mechanisms [9]. It is clear that diabetes leads to a relative increase in accumulation of myeloid cells in the artery wall, in both atherosclerosis progression models like the one used in the present study and in atherosclerosis regression models [9]. The mechanism appears to involve altered fatty acid handling in myeloid cells [3], increased activation of the receptor for advanced endproducts [9, 56], increased oxidative stress through NADPH oxidase 1 [57], increased cholesterol accumulation in bone marrow progenitor cells [58], and most likely increased adhesion molecule expression by endothelial cells [59, 60]. While the current study adds an important missing piece to the puzzle, further studies are needed to elucidate the mechanisms of diabetes-accelerated atherogenesis.

In summary, in this mouse model of T1DM increased myeloid cell PGE_2_-EP4 signaling contributes significantly to some aspects of diabetes-exacerbated inflammation, but does not alter atherosclerosis.

## Abbreviations

BMDC: bone marrow-derived dendritic cell
BMDM: bone marrow-derived macrophage
EP: prostaglandin receptor
IL: interleukin
LPS: lipopolysaccharide
PGE_2_: prostaglandin E_2_
T1DM: type 1 diabetes mellitus
T2DM: type 2 diabetes mellitus
WBC: white blood cell
WT: wildtype
